# Acute toxicity study of tilmicosin-loaded hydrogenated castor oil-solid lipid nanoparticles

**DOI:** 10.1186/1743-8977-8-33

**Published:** 2011-11-20

**Authors:** Shuyu Xie, Fenghua Wang, Yan Wang, Luyan Zhu, Zhao Dong, Xiaofang Wang, Xihe Li, WenZhong Zhou

**Affiliations:** 1 Department of Preventive Veterinary Medicine, College of Veterinary Medicine, China Agricultural University, 2 Yuanmingyuan Road West, Beijing 100193, PR China; 2 Inner Mongolia Saikexing Reproductive Biotechnology Co. Ltd./Inner Mongolia University College of Life Sciences, Mengniu Dary R&D Center, Shengle Economic District, Helin Geer, Huhhot, Inner Mongolia 011517, PR China

**Keywords:** Tilmicosin, hydrogenated castor oil (HCO), solid lipid nanoparticles (SLN), acute toxicity

## Abstract

**Background:**

Our previous studies demonstrated that tilmicosin-loaded hydrogenated castor oil solid lipid nanoparticles (Til-HCO-SLN) are a promising formulation for enhanced pharmacological activity and therapeutic efficacy in veterinary use. The purpose of this work was to evaluate the acute toxicity of Til-HCO-SLN.

**Methods:**

Two nanoparticle doses were used for the study in ICR mice. The low dose (766 mg/kg.bw) with tilmicosin 7.5 times of the clinic dosage and below the median lethal dose (LD_50_) was subcutaneously administered twice on the first and 7th day. The single high dose (5 g/kg.bw) was the practical upper limit in an acute toxicity study and was administered subcutaneously on the first day. Blank HCO-SLN, native tilmicosin, and saline solution were included as controls. After medication, animals were monitored over 14 days, and then necropsied. Signs of toxicity were evaluated via mortality, symptoms of treatment effect, gross and microscopic pathology, and hematologic and biochemical parameters.

**Results:**

After administration of native tilmicosin, all mice died within 2 h in the high dose group, in the low dose group 3 died after the first and 2 died after the second injections. The surviving mice in the tilmicosin low dose group showed hypoactivity, accelerated breath, gloomy spirit and lethargy. In contrast, all mice in Til-HCO-SLN and blank HCO-SLN groups survived at both low and high doses. The high nanoparticle dose induced transient clinical symptoms of treatment effect such as transient reversible action retardation, anorexy and gloomy spirit, increased spleen and liver coefficients and decreased heart coefficients, microscopic pathological changes of liver, spleen and heart, and minor changes in hematologic and biochemical parameters, but no adverse effects were observed in the nanoparticle low dose group.

**Conclusions:**

The results revealed that the LD_50 _of Til-HCO-SLN and blank HCO-SLN exceeded 5 g/kg.bw and thus the nanoparticles are considered low toxic according to the toxicity categories of chemicals. Moreover, HCO-SLN significantly decreased the toxicity of tilmicosin. Normal clinic dosage of Til-HCO-SLN is safe as evaluated by acute toxicity.

## Background

Tilmicosin is a semi-synthetic macrolide antibiotic developed for veterinary use with properties that include low inhibitory concentration, broad antimicrobial spectrum, a large distribution volume, long elimination half-life and rapid accumulation in bovine macrophages and mammary gland epithelial cells [1,2]. Rapid and extensive penetration of tilmicosin from blood into milk, and slow elimination from the milk were among the characteristic kinetic features of the drug after intravenous and subcutaneous administration [1]. However, acute cardiac toxicity following intravenous injection was serious [1]. With these characteristics, subcutaneous administration of tilmicosin would be ideal for the treatment of bovine mastitis. However, subcutaneous administration of tilmicosin was much less effective as a dry cow or lactating cow therapeutics against *Staphylococcus aureus *mastitis [3,4]. The ineffectiveness of tilmicosin against infections may be related to inadequate therapeutic drug concentrations and/or an insufficient residence time of the antibiotic in the mammary gland [5]. Repeated administrations and high dose could enhance the clinical efficacy, but high doses of tilmicosin can result in acute cardiac toxicity in cattle and severe reactions at the injection sites such as vascular collapse and transient swelling caused by a transient high drug concentration [6,7]. The severity of the side effects is dose-dependent [8].

Our previous studies demonstrated that the tilmicosin-loaded hydrogenated castor oil solid lipid nanoparticles (Til-HCO-SLN) are a promising formulation to enhance the pharmacological activity and therapeutic efficacy of tilmicosin, and can decrease reactions at injection sites [5,9]. Pharmacokinetics study after subcutaneous administration to mice demonstrated that SLN significantly extended systemic circulation time, increased the bioavailability, and decreased the maximum serum concentration of the drug [9]. Therapeutic study in a mouse mastitis model showed that the SLN enhanced the antibacterial activity and therapeutic efficacy of tilmicosin with reduced dose and frequency of administration [5]. Microscopic pathology study demonstrated that SLN did not induce inflammation at the injection site [9].

Although it is well known that SLN have good biocompatibility, biodegradability and tolerability, there are limited reports on their toxicity. The available data revealed that the toxicity of SLN depends on their physical dimensions (such as size and shape), lipid matrix, incorporated drug and surface chemistry [10-12]. Hydrogenated castor oil (HCO) has excellent tolerability, but HCO formulated nanoparticles could be different. The specific physicochemical properties of nanoparticles such as surface properties, chemical reactivity, physical absorption ability and permeability are different from those of the same composition bulk materials and such properties might result in undesirable effects [13].

In this study, the acute toxicity of Til-HCO-SLN was investigated in ICR mice via mortality, clinical status, food and water consumption, body weight, gross and microscopic pathology, and hematologic and biochemical parameters.

## Materials and methods

### Materials

Hydrogenated castor oil was purchased from Tongliao Tonghua Castor Chemical Co., Ltd. Tilmicosin was obtained from Jining Medicine Corporation. Polyvinyl alcohol (PVA) was purchased from Sigma (St. Louis, MO, USA). HCO was used as lipid matrix and PVA was utilized as emulsifier to prepare the nanoparticles by a hot homogenization and ultrasonication method as described previously [5]. The encapsulation efficiency and loading capacity of Til-HCO-SLN was determined using a UV spectrophotometer method as described previously [5]. The diameter, polydispersivity index and zeta potential analysis of the nanoparticles was performed by photon correlation spectroscopy using Zetasizer Nano ZS90 (Malvern Instruments) [5]. The residual PVA of Til-HCO-SLN was determined by a colorimetric method [9]. The encapsulation efficiency, loading capacity, diameter, polydispersivity index, zeta potential and residual PVA of Til-HCO-SLN were 59.82 ± 5.16%, 9.97 ± 0.86%, 334 ± 44 nm, 0.49 ± 0.04, -5.72 ± 0.34 mV and 0.35 ± 0.03%, respectively. Blank HCO-SLN had particle size 369 ± 54 nm, PDI 0.35 ± 0.05, zeta potential -11.46 ± 1.01 mV, and residual PVA 0.32 ± 0.04%. Other chemicals and reagents not specified in the text were of analytical grade or equivalent.

### Animals

ICR mice of each sex (20-22 g) were obtained from Vital River Beijing (Beijing, China). The animals were housed at room temperature under natural day and night cycles with free access to water and food. They were kept for one week before use. All experimental protocols concerning the handling of mice were in accordance with the requirements of the Institutional Animal Care and Use Committee at China Agricultural University.

### Experimental design

Eighty mice were randomly divided into 8 groups with 5 male and 5 female animals in each group. Two SLN doses were used in the experiment. The high dose of 5 g/kg.bw (equivalent to tilmicosin 540 mg/kg.bw) was given once on the first day. The low dose of 766 mg/kg.bw (equivalent to tilmicosin 75 mg/kg.bw) was given twice on the first and 7th day. The Til-HCO-SLN was suspended in 500 μl sterilized saline and subcutaneously administered on the dorsum of mice. The same amount of blank HCO-SLN and native tilmicosin suspended or dissolved in 500 μl sterilized saline and also the same volume of sterilized saline were subcutaneously administered in different control groups.

### Clinical sign observation

After medication, the mice were observed over a 14 day period. The symptoms of toxicity such as mortality, spirit, behavior, and appetite as well as body weight were record daily.

### Necropsy

At the end of the experiment, blood samples were collected from the eye vein for biochemical and hematological assays. Then the animals were necropsied to evaluate the gross internal abnormalities viz. organ coefficients (relative organ weight) and appearance of heart, lung, liver, spleen and kidneys. The organ coefficients were calculated [14]:

$$\text{Organ coefficients }=\left(\text{Weight of organ }\left(\text{g}\right)∕\text{Body weight }\left(\text{g}\right)\right)\;\times \text{ 1}00\%$$

### Histopathological study

The heart, liver, lung, spleen and kidneys were fixed in 4% formalin and embedded in paraffin. Sections of 5 μm thick were cut and placed onto glass slides. After staining with hematoxylin and eosin, histopathological changes were observed using optical microscope (Olympus IX71, Japan).

### Biochemical assays

The blood samples were centrifuged at 3000 rpm for 10 min using Sigma centrifuge (Sigma1-14, Germany). Serum levels of alanine aminotransferas (ALT), aspartate aminotransferase (AST), alkaline phosphatase (ALP), cholesterol esters (CHOL), total proteins (TP), albumin (ALB), globulin (GLB), total bilirubin (TBIL), blood urea nitrogen (BUN), creatinine (CREA) and glucose (GLU) were measured with a biochemical autoanalyzer (Type 7170, Hitachi, Japan) [15,16].

### Hematological study

Levels of white blood cells (WBC), red blood cells (RBC), haematocrit (HCT), mean corpuscular volume (MCV), hemoglobin (HGB), mean corpuscular haemoglobin (MCH), mean corpuscular haemoglobin concentration (MCHC), neutrophils (NEU), lymphocytes(LYM), monocytes (MON), eosinophils (EOS), basophils (BAS), blood platelet (PLT), plateletcrit (PCT), mean platelet volume (MPV) and red cell distribution width (RDW) were determined by an automatic hematology analyzer (ABX, Pentra 60, France) [17,18].

### Statistical analysis

Results were expressed as mean ± standard deviation (S.D.) and were compared by one-way analysis of variance (ANOVA) using Statistical Product and Service Solutions (SPSS) V13.0 (SPSS Inc., Chicago, IL, USA). The differences were considered statistically significant at the level of P < 0.05.

## Results

### Clinical signs and mortality

Table 1 shows the ratio of animals with clinical signs and the mortality after the different treatments. After administration with the high dose, all mice treated with native tilmicosin died within 2 h. In contrast, no mice died in Til-HCO-SLN group during the observed period. There were no obvious adverse responses except for the transient reversible action retardation, anorexy and gloomy spirit within 48 h. In the blank HCO-SLN group, all mice survived and the clinical signs were similar to those in the Til-HCO-SLN group.

**Table 1:** The ratio of animals with clinical signs and the mortality after different treatment

Compound	Dose (mg/kg.bw) SLN/Tilmicosin	Animal number (Male/Female)	Animals with clinical sign/treated animals		Died animals/treated animals	
			Male	Female	Male	Female
Til-HCO-SLN	5000/540	5/5	5/5	5/5	0/5	0/5
Til-HCO-SLN*	766/75	5/5	0/5ab	0/5ab	0/5ab	0/5ab
HCO-SLN	5000/0	5/5	5/5	5/5	0/5	0/5
HCO-SLN*	766/0	5/5	0/5ab	0/5ab	0/5ab	0/5ab
Tilmicosin	-/540	5/5	5/5	5/5	5/5	5/5
Tilmicosin*	-/75	5/5	5/5a, 3/3b	5/5a, 4/4b	2/5a, 1/3b	1/5a, 1/4b
Saline	-/-	5/5	0/5	0/5	0/5	0/5
Saline*	-/-	5/5	0/5ab	0/5ab	0/5ab	0/5ab

* Animal treated twice on the first and 7th day: (a) after first injection; (b) after second injection.

At the low dose, Til-HCO-SLN and blank HCO-SLN did not cause mortality and any apparent toxicity such as abnormal spirit and behaviors. The low dose of native tilmicosin induced action retardation, incoordination, accelerated breath and gloomy spirit immediately after the first administration, and two male and one female died within 2 h. The spirit and behavior of surviving mice were gradually recovered. Ten min after the second administration, the mice displayed accelerated breath and gloomy spirit, and stayed together, not willing to move. One male and one female mouse died within 2 h and the survived mice recovered gradually.

### Growth rate

**Figure 1:**
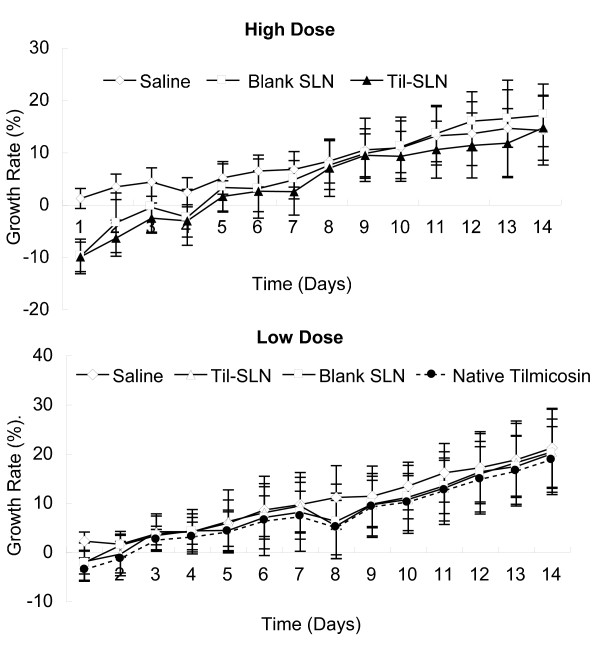
**Cumulative growth rates of mice after treatment**.

Figure 1 shows the cumulative growth rates of mice. At the high dose, the cumulative growth rate of Til-HCO-SLN group was negative and significantly lower than the saline control group within 3 days post treatment. Three days later, the growth rate increased to the same level as the saline control group. Similar results were obtained in blank HCO-SLN group. At the low dose, no significant differences of cumulative growth rates were found in Til-HCO-SLN, blank HCO-SLN, native tilmicosin and saline groups.

### Food and water consumption

**Figure 2:**
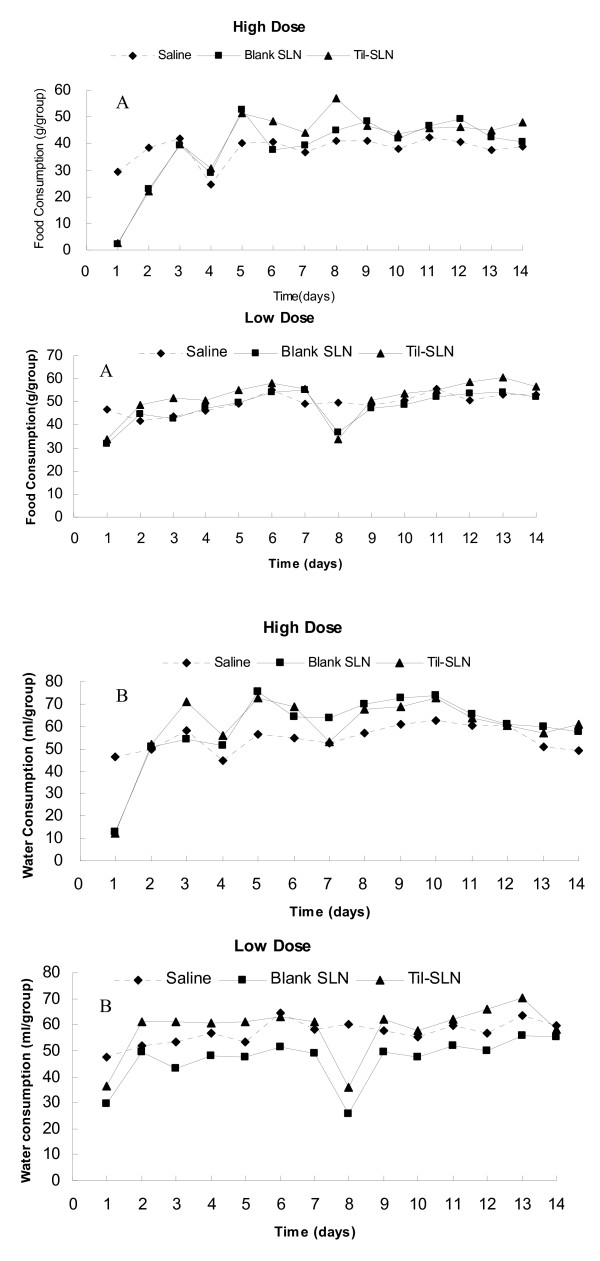
**Daily food and water consumption of mice after treatment**. A: Daily food consumption; B: Daily water consumption.

Daily food and water consumption is shown in Figure 2. After administration of the high dose, the mice in Til-HCO-SLN and blank HCO-SLN groups hardly eat food or drink water during the first day, but they gradually recovered foraging. Daily water and food consumption completely recovered by the second and fifth day, respectively.

With the low dose medication, the adverse effect of reduced food intake was similar as for high dose but to a lower extent. Daily food and water consumption in Til-HCO-SLN group was much similar to that in blank HCO-SLN group. The two nanoparticle groups showed almost the same levels of daily food and water consumption as the control saline group except for the 1st and 8th injection days on which the food and water consumption was less compared with the saline control group.

### Organ appearances and coefficients

Necropsy at the end of the experiment (day 14) did not show any macroscopic organ changes in Til-HCO-SLN and other control groups with either high or low doses. With the high dose, slight increases of liver and spleen coefficients were observed in Til-HCO-SLN and blank HCO-SLN groups, while the heart coefficient was slightly decreased in Til-HCO-SLN group (Table 2).

**Table 2:** Coefficients of heart, liver, spleen, lung and kidneys (Mean ± S.D., n = 10).

Compound		Heart	Liver	Spleen	Lung	Kidneys
Saline	H	0.602 ± 0.134	4.536 ± 0.632	0.333 ± 0.063	0.657 ± 0.105	1.333 ± 0.134
	L	0.594 ± 0.051	5.038 ± 0.534	0.368 ± 0.075	0.720 ± 0.121	1.689 ± 0.271
Blank HCO-SLN	H	0.583 ± 0.070	5.063 ± 0.453^b^	0.447 ± 0.124^b^	0.626 ± 0.109	1.364 ± 0.214
	L	0.642 ± 0.067	4.787 ± 0.813	0.426 ± 0.045	0.732 ± 0.161	1.661 ± 0.420
Til-HCO-SLN	H	0.477 ± 0.076^a^	5.072 ± 0.493^a^	0.425 ± 0.100^a^	0.629 ± 0.147	1.305 ± 0.226
	L	0.618 ± 0.076	4.405 ± 0.877	0.395 ± 0.075	0.732 ± 0.119	1.659 ± 0.222
Native Tilmicosin	L	0.662 ± 0.030	4.740 ± 0.600	0.359 ± 0.076	0.707 ± 0.078	1.548 ± 0.192

^a ^Significant difference between Til-HCO-SLN and saline treated mice, p < 0.05.

^b ^Significant difference between blank HCO-SLN and saline treated mice, p < 0.05.

H: high dose; L: low dose.

With the low dose, the coefficients of heart, liver, spleen, lung and kidneys were not significantly different among the Til-HCO-SLN, blank HCO-SLN, native tilmicosin and saline groups (Table 2).

### Histopathology

**Figure 3:**
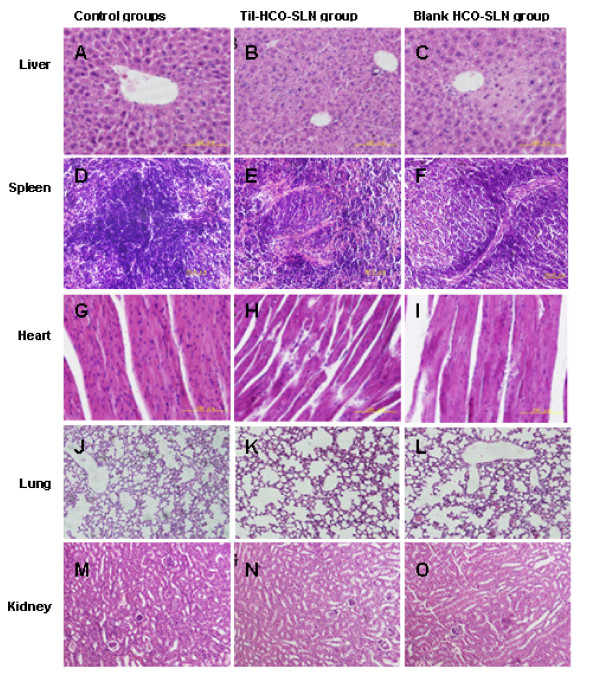
**Organ histopathological picture of mice treat with the high dose (H&E, magnification ×200)**. Liver histopathological pictures of mice treated with sterilized saline (A), Til-HCO-SLN (B) and blank HCO-SLN (C); Spleen histopathological pictures of mice treated with sterilized saline (D), Til-HCO-SLN (E) and blank HCO-SLN (F); Heart histopathological pictures of mice treated with sterilized saline (G), Til-HCO-SLN (H) and blank HCO-SLN (I); Lung histopathological pictures of mice treated with sterilized saline (J), Til-HCO-SLN (K) and blank HCO-SLN (L); Kidney histopathological pictures of mice treated with sterilized saline (M), Til-HCO-SLN (N) and blank HCO-SLN (O).

With the high dose, both Til-HCO-SLN and blank HCO-SLN induced histopathological changes in liver, spleen and heart, while the changes in blank HCO-SLN group were slight (Figure 3). The histopathology alteration in liver includes swelling, degeneration, necrosis of certain amounts of hepatocytes around the central vein, and obscure lobular boundaries. The spleen displayed dwindling of splenic corpuscle and reducing of the splenic lymphocytes. Degeneration and necrosis of cardiac cells and necrosis and breakage of cardiac muscle fibers were observed in the heart.

**Figure 4:**
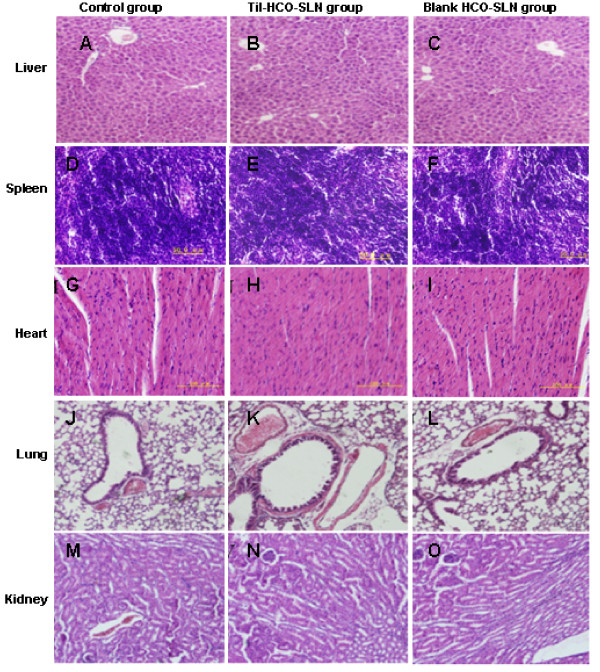
**Organ histopathological picture of mice treat with the low dose (H&E, magnification ×200)**. Liver histopathological pictures of mice treated with sterilized saline (A), Til-HCO-SLN (B) and blank HCO-SLN (C); Spleen histopathological pictures of mice treated with sterilized saline (D), Til-HCO-SLN (E) and blank HCO-SLN (F); Heart histopathological pictures of mice treated with sterilized saline (G), Til-HCO-SLN (H) and blank HCO-SLN (I); Lung histopathological pictures of mice treated with sterilized saline (J), Til-HCO-SLN (K) and blank HCO-SLN (L); Kidney histopathological pictures of mice treated with sterilized saline (M), Til-HCO-SLN (N) and blank HCO-SLN (O).

After low dose injection, no observable pathological changes were found in heart, liver, lung, kidneys and spleen (Figure 4).

### Biochemistry index

Biochemistry study showed that at the high dose there were some changes of some biochemical parameters in both the Til-HCO-SLN and blank HCO-SLN groups compared with the saline group, but the biochemical parameter values were at similar levels and the differences were very slight (Table 3). At the low dose, no statistical change of biochemical parameters was observed in Til-HCO-SLN treated, as well as blank HCO-SLN and native tilmicosin treated mice compared with saline treated controls (Table 3).

**Table 3:** Biochemical parameters of treated mice (Mean ± S.D., n = 10).

Parameters		Saline	Til-HCO-SLN	Blank HCO-SLN	Tilmicosin (n = 5)
AST (U/L)	H	147.78 ± 37.78	157.20 ± 34.95	150.20 ± 24.15	
	L	152.14 ± 18.77	143.29 ± 19.79	150.00 ± 16.12	171.00 ± 17.15
ALT (U/L)	H	44.56 ± 17.37	37.11 ± 9.18	41.00 ± 10.79	
	L	42.86 ± 8.28	47.14 ± 11.05	49.57 ± 7.28	50.75 ± 0.96
ALT/AST	H	0.30 ± 0.04	0.26 ± 0.06	0.27 ± 0.05	
	L	0.29 ± 0.06	0.33 ± 0.07	0.33 ± 0.05	0.30 ± 0.03
ALP (U/L)	H	136.44 ± 34.46	150.60 ± 19.57	140.29 ± 21.47	
	L	121.71 ± 22.89	111.14 ± 19.30	121.50 ± 27.77	135.25 ± 17.00
TBIL (umol/L)	H	2.56 ± 1.43	1.86 ± 0.94	1.81 ± 0.89	
	L	0.90 ± 0.42	0.74 ± 0.35	1.09 ± 0.41	1.38 ± 1.05
CHOL (mmol/L)	H	3.86 ± 0.48	4.21 ± 0.89	3.81 ± 0.92	
	L	3.53 ± 0.54	3.54 ± 0.62	3.69 ± 0.74	3.67 ± 0.44
CREA (umol/L)	H	11.60 ± 3.27	13.13 ± 1.73	11.80 ± 2.53	
	L	18.29 ± 4.57	18.00 ± 3.42	18.25 ± 1.91	17.25 ± 0.96
BUN (mmol/L)	H	7.53 ± 1.96	7.00 ± 1.12	6.72 ± 1.11	
	L	8.59 ± 1.54	9.54 ± 1.48	9.47 ± 1.79	9.70 ± 0.58
TP (g/L)	H	60.90 ± 2.77	60.80 ± 2.57	56.00 ± 3.02^b^	
	L	61.31 ± 3.57	59.37 ± 1.86	60.69 ± 2.81	61.03 ± 2.16
ALB (g/L)	H	41.49 ± 1.60	40.44 ± 2.13	38.34 ± 1.26^b^	
	L	38.09 ± 4.89	37.06 ± 5.21	36.56 ± 3.35	38.90 ± 4.22
GLB (g/L)	H	19.41 ± 1.45	20.36 ± 1.31	17.66 ± 2.01^b^	
	L	23.21 ± 2.18	22.31 ± 3.94	24.13 ± 2.70	22.13 ± 2.81
ALB/GLB	H	2.14 ± 0.12	1.99 ± 0.16^a^	2.19 ± 0.19	
	L	1.71 ± 0.34	1.84 ± 0.46	1.74 ± 0.31	1.80 ± 0.41
GLU (mmol/L)	H	6.07 ± 1.68	5.98 ± 1.27	6.33 ± 1.45	
	L	5.18 ± 1.78	5.02 ± 1.51	4.95 ± 1.18	5.78 ± 0.57

^a ^Significant difference between Til-HCO-SLN and saline treated mice, p < 0.05.

^b ^Significant difference between blank HCO-SLN and saline treated mice, p < 0.05.

**Abbreviations: **H: high dose; L: low dose; AST: aspartate aminotransferase; ALT: serum alanine aminotransferas; ALP: alkaline phosphatase; TBIL: total bilirubin; CHOL: cholesterol esters; CREA: creatinine; BUN: blood urea nitrogen; TP: total proteins; ALB: albumin; GLB: globulin; GLU: glucose.

### Hematology index

The hematological parameters in Til-HCO-SLN group had no obvious changes compared with the saline control group with both the high and low doses (Table 4). At the high dose, there were some statistical differences of hematological parameters between the blank HCO-SLN and saline groups, but the differences were slight (Table 4).

**Table 4:** Hematological parameters of treated mice (Mean ± S.D., n = 10).

Parameters		Saline	Til-HCO-SLN	Blank HCO-SLN	Tilmicosin (n = 5)
WBC (10^9^/L)	H	5.51 ± 1.55	5.52 ± 1.68	4.95 ± 1.39	
	L	5.89 ± 1.80	5.05 ± 1.06	5.22 ± 0.93	5.16 ± 0.78
RBC	H	8.56 ± 1.30	7.58 ± 1.40	7.07 ± 1.14^b^	
(10^12^/L)	L	9.64 ± 0.79	9.58 ± 0.96	9.63 ± 0.56	9.08 ± 0.69
HCT (L/L)	H	0.44 ± 0.05	0.40 ± 0.03	0.36 ± 0.05^b^	
	L	0.59 ± 0.09	0.61 ± 0.07	0.58 ± 0.06	0.57 ± 0.07
MCV (fL)	H	51.01 ± 2.69	51.06 ± 2.10	51.72 ± 4.05	
	L	60.37 ± 5.74	63.18 ± 4.22	60.63 ± 5.34	62.47 ± 4.76
HGB (g/L)	H	141.44 ± 10.48	129.89 ± 10.25	119.43 ± 15.80^b^	
	L	153.63 ± 9.97	155.00 ± 12.00	153.38 ± 6.02	147.80 ± 4.21
MCH (pg)	H	16.84 ± 2.33	16.44 ± 1.67	16.99 ± 1.77	
	L	15.96 ± 0.46	16.21 ± 0.57	15.94 ± 0.54	16.30 ± 0.80
MCHC (g/L)	H	329.33 ± 32.11	320.70 ± 22.14	328.00 ± 19.47	
	L	266.88 ± 29.78	257.13 ± 15.75	264.50 ± 23.68	262.40 ± 27.93
PLT (10^9^/L)	H	873.89 ± 139.28	812.29 ± 157.35	802.00 ± 154.55	
	L	971.13 ± 145.25	967.63 ± 171.96	925.88 ± 219.88	972.60 ± 233.96
MPV (fL)	H	6.74 ± 1.78	6.50 ± 1.14	6.25 ± 1.16	
	L	5.43 ± 0.29	5.28 ± 0.21	5.25 ± 0.22	5.32 ± 0.38
PCT (L/L)	H	0.57 ± 0.21	0.47 ± 0.10	0.40 ± 0.11^b^	
	L	0.53 ± 0.07	0.57 ± 0.09	0.55 ± 0.11	0.56 ± 0.06
RDW (fL)	H	11.83 ± 3.81	11.70 ± 2.28	11.75 ± 1.74	
	L	17.21 ± 0.79	18.00 ± 1.90	17.47 ± 2.02	17.72 ± 1.37
NEU (%)	H	14.75 ± 2.02	15.70 ± 2.22	17.83 ± 1.50	
	L	15.96 ± 5.04	20.06 ± 5.33	18.99 ± 3.12	20.44 ± 5.95
LYM (%)	H	84.25 ± 2.78	81.64 ± 3.94	80.63 ± 1.32	
	L	80.71 ± 5.68	74.93 ± 5.84	76.43 ± 4.69	76.88 ± 6.40
MON (%)	H	0.85 ± 0.07	1.73 ± 1.13	1.30 ± 0.36	
	L	2.84 ± 1.03	4.30 ± 1.77	3.85 ± 1.73	2.24 ± 1.18
EOS (%)	H	0.20 ± 0.08	0.23 ± 0.19	0.15 ± 0.10	
	L	0.26 ± 0.17	0.40 ± 0.13	0.33 ± 0.10	0.16 ± 0.11
BAS (%)	H	0.17 ± 0.10	0.24 ± 0.14	0.18 ± 0.16	
	L	0.23 ± 0.07	0.31 ± 0.22	0.41 ± 0.29	0.28 ± 0.15

^a ^Significant difference between Til-HCO-SLN and saline treated mice, p < 0.05.

^b ^Significant difference between blank HCO-SLN and saline treated mice, p < 0.05.

**Abbreviations: **H: high dose; L: low dose; WBC: white blood cells; RBC: red blood cells; HCT: haematocrit; MCV: mean corpuscular volume; HGB: hemoglobin; MCH: mean corpuscular haemoglobin; MCHC: mean corpuscular haemoglobin concentration; PLT: blood platelet; MPV: mean platelet volume; PCT: plateletcrit; RDW: red cell distribution width; NEU: neutrophils; LYM: lymphocytes; MON: monocytes; EOS: eosinophils; BAS: basophils.

## Discussion

Acute toxicity values such as LD_50 _and symptoms of treatment effect are often used as the basis for classifying chemicals into toxicity categories, and their subsequent regulation [19,20]. In this study, two nanoparticle doses were used to evaluate the acute toxicity of Til-HCO-SLN. The high dose was 5 g/kg b.w, which is the highest dose in an acute toxicity study according to the guidelines for toxicity testing [20,21]. The low nanoparticle dose of 766 mg/kg.bw, equivalent to 75 mg/kg.bw tilmicosin, was chosen based on the clinic dosage and the median lethal dose (LD_50_). If the overdose did not induce treatment effect, the clinic dosage should be safe. At the same time, the main focus of the low dose was on observing the treatment symptoms rather than on determining the mortality, and thus the dose was below the LD_50°_The LD_50 _of tilmicosin for subcutaneous administration is 97 mg/kg in male ICR mice and 109 mg/kg.bw in female ICR mice [22].

The results showed that Til-HCO-SLN and blank HCO-SLN was not lethal to mice with the high dose, suggesting that the Til-HCO-SLN and blank HCO-SLN are classified as low-toxic substances according to the toxicity categories of chemicals [23]. This is mainly attributed to the good biocompatibility and biodegradability of the lipid matrix and the emulsifier. HCO can be hydrolyzed in vivo resulting in the formation of fatty acid and glycerin as metabolites [24]. PVA can be degraded into acetic acid by oxidase and hydrolase enzyme [25]. The oral LD_50 _is in the range of 15-20 g/kg [25], while the residual PVA of Til-HCO-SLN and blank HCO-SLN was very low.

The high dose of Til-HCO-SLN and blank HCO-SLN induced some slight intoxication signs of transient reversible abnormal clinic symptoms. The liver and spleen were affected as indicated by the organ coefficients and pathological changes, which is in agreement with other report that high doses of SLN could lead to accumulation of the lipid in liver and spleen and subsequently to pathological alterations [10]. This might be due to that liver and spleen are the two dominant organs for distribution and metabolism of SLN [26,27]. The damage of liver function is also confirmed by some biochemical parameter changes. Til-HCO-SLN caused slight decrease of the albumin (ALB)/globulin (GLB), while blank HCO-SLN leads to minor reduction of ALB and GLB. The hepatocellular damage is often indicated by changes of albumin, globulins and total protein [28]. However, other liver function related parameters such as alanine aminotransferas (ALT), aspartate aminotransferase (AST), ALT/AST, bilirubin and urea were normal, suggesting that the damage of liver was slight and reversible [26]. In addition, Til-HCO-SLN induced heart coefficient and histopathological changes, while blank HCO-SLN resulted in no coefficient change and much less histopathological alteration. This could be due to the acute cardiac toxicity of tilmicosin [22].

With low dose, the mice in the Til-HCO-SLN and blank HCO-SLN groups did not display any intoxication syndrome as referenced with the high dose groups. All mice survived and showed no clinic symptoms of treatment. There were no changes of organ appearance, organ coefficient, histopathology, biochemical and haematological parameters. The transient decrease of daily food and water consumption after the injections on the 1st and 8th days could be due to the irritation of the drug [29]. These results indicate that the nanoparticle carrier and the formulation were safe at 7.5 times of the clinic dosage.

After administration with native tilmicosin, all mice in the high dose group died within 2 h. The surviving mice in the low dose group showed action retardation, incoordination, accelerated breath and gloomy spirit within 2 h after each administration. Tilmicosin primarily affects the heart, leading to acute heart failure with clinical signs of depression, increased heart rate, pulmonary edema, and death [22,30]. Tilmicosin did not influence the organ appearance and coefficients, biochemical and hematological parameters at the low dose. The intoxication symptoms could be too low to observe. Another reason might be that the acute toxicity of tilmicosin was temporary [31], and all the functions were recovered at the end of experiments. In contrast, all mice survived and showed no treatment adverse effect in Til-HCO-SLN group with the low dose. These results demonstrated that this nanoparticle carrier remarkably reduced the acute toxicity of tilmicosin. The lower transient drug concentration of Til-HCO-SLN could account for the reduction of acute toxicity of tilmicosin [7,8].

## Conclusion

The HCO-SLN is low toxic judged by the LD_50 _although clinical symptoms indicated some toxicity_. _This nanoparticle carrier can significantly decrease the toxicity of tilmicosin. Normal clinic dosage of Til-HCO-SLN is safe as evaluated by acute toxicity. The subchronic and chronic toxicity of Til-HCO-SLN need to be further studied.

## List of abbreviations

Til-HCO-SLN: tilmicosin-loaded hydrogenated castor oil solid lipid nanoparticles; LD_50_: median lethal dose; SLN: solid lipid nanoparticles; HCO: hydrogenated castor oil; PVA: Polyvinyl alcohol; AST: aspartate aminotransferase; ALT: serum alanine aminotransferas; ALP: alkaline phosphatase; TBIL: total bilirubin; CHOL: cholesterol esters; CREA: creatinine; BUN: blood urea nitrogen; TP: total proteins; ALB: albumin; GLB: globulin; GLU: glucose; WBC: white blood cells; RBC: red blood cells; HCT: haematocrit; MCV: mean corpuscular volume; HGB: hemoglobin; MCH: mean corpuscular haemoglobin; MCHC: mean corpuscular haemoglobin concentration; PLT: blood platelet; MPV: mean platelet volume; PCT: plateletcrit; RDW: red cell distribution width; NEU: neutrophils; LYM: lymphocytes; MON: monocytes; EOS: eosinophils; BAS: basophils.

## Competing interests

The authors declare that they have no competing interests.

## Authors' contributions

WZZ, SYX and FHW designed the study. FHW performed the experiments. SYX and FHW collected and analyzed data. WZZ and SYX wrote the manuscript. Other authors gave technical support and conceptual advice. All authors read and approved the final manuscript.
